# Impact of Advertising Campaigns Among Online Gamblers: The Role Perceptions of Social Support and Personality Traits

**DOI:** 10.3389/fpsyt.2021.599988

**Published:** 2021-10-26

**Authors:** Samantha Tessier, Lucia Romo, Oulmann Zerhouni

**Affiliations:** ^1^Département de Psychologie, Clinique Psychanalyse Développement, Nanterre, France; ^2^AP-HP (Paris Hospital), Occupational Health Unit, Poincaré University Hospital, Garches, France; ^3^Département de Psychologie, Laboratoire Parisien de Psychologie Sociale, Nanterre, France

**Keywords:** social support, big five model, personality, gambling advertisements, online gambling

## Abstract

**Background:** Few studies on problematic gamblers have focused on how environment and personality interact in gambling behavior. The aim of this research is to investigate how social support, dimensions of personality, and advertising campaigns are associated with gambling among problematic or moderate-risk gamblers and recreational gamblers and associated with online gambling (i.e., sport and poker).

**Methods:** One hundred nine participants (45% problematic or moderate-risk gamblers) answered an online survey including social support, five factor models of personality, typology of gamblers, and several sociodemographic variables.

**Results:** We found that problematic and moderate-risk gamblers were significantly more sensitive to gambling advertisements compared to light players. Social support was significantly lower among online gamblers compared to offline gamblers, but no association was found between social support and type of gamblers. Problematic and moderate-risk gamblers presented lower levels of extraversion compared with recreational gamblers. Notably, when the onset of gambling is before 18 years old, participants had more chances to recall more gambling advertisements as adults.

**Conclusion:** We propose that future longitudinal research should focus on characteristics of online gamers particularly regarding social support to understand this low level of adequacy compared to offline gamblers.

## Introduction

### Filling the Gap About Cognitive Antecedents of Advertising Influence

Gambling encompasses a variety of games, from gaming machines, casino gambling, lotteries, poker, animals, to sports betting. In addition, the Diagnostic and Statistical Manual of Mental Disorders, Fifth Edition [DSM-5, (1)] reclassified gambling disorder as a pathology, indicating a better identification of the phenomenon and his importance. In France in 2020, a study in the general population indicated that 74% gambled at least one time during their life and 47% in the last 12 months (2). Few studies have been conducted on the impact of advertising for gambling, but as a first approximation [see (3)], parallels can be drawn with advertising for other types of substances [see for example, (4)]. For example, studies on tobacco and alcohol showed that greater exposure to advertising is associated with more positive attitudes, intentions, and actual consumption (5). Adolescents seem particularly receptive to gambling campaigns. Minors report receiving numerous emails promoting the game; they recall television campaigns, and non-gamblers may be encouraged to gamble (6, 7). Advertising gambling campaigns on social media or mainstream media produce the same behaviors among young people (8). Among adults, recreational gamblers are less influenced by advertising campaigns than problem gamblers (8). Among problem gamblers, a Swedish study indicates that 25% of them felt a strong incentive to gamble after watching campaigns, and 50% felt a moderate incentive. Nonetheless, the study does find that gambling campaigns trigger impulsiveness to gamble (9). Promotional offers appear to be a factor that increases the incentive to gamble for all players. While these offers do not appear to drive recreational gamblers toward problem gambling (10), problem gamblers indicate that these promotional offers increase their gambling problems (11). Moreover, looking at long-term memory and declaration of recollection, discordant results are found in the literature: a correlation is sometimes found between the recall of advertising campaigns and gambling severity (12, 13) and sometimes not (10).

Hence, the bulk of these studies have focused on external determinants—such as the advertising environment—of gambling (3). To date, few studies focused on the relationship between exposure to gambling advertising and gambling attitudes, intentions, and behavior, but rather focused on gambling intentions. In this article, we provide novel, self-reported, observational data on how internal, self-regulatory factors influence gambling, that is (i) subjective, perceived social support and (ii) personality traits of gamblers on the severity of their gambling addiction and sensitivity to gambling advertising. All data was collected online.

### Social Support and Personality Factors as Self-Regulatory Factors in Gambling

Self-regulation is defined as the ability to regulate emotional, cognitive, and behavioral responses, allowing individuals to select the most appropriate responses to external demands. Research shows that cognitive processing of emotional stimuli is involved in the etiology and maintenance of various psychopathologies. For example, anxiety is associated with an attentional bias toward threatening stimuli (14), and a decreased ability to self-regulate is associated with chronic anxiety (15) and the maintenance of addictive behavior (16). Thus, differences in self-regulatory abilities are likely to be involved in the perception and recall of advertisements representing relevant, appetitive stimuli for the participant [see (17–19)].

Here, we will focus on two factors influencing self-regulation: social support, in that it contributes to effective emotional regulation, and the influence of personality traits, particularly traits involved in emotional feeling (i.e., neuroticism, extraversion, and agreeableness).

Social support could be defined as the connections that individuals have with significant non-professional others in their social environments, the perceived social support resulting from the cognitive appraisal of being reliably connected to others, or the assistance that others realize when they help other people (20, 21). Social support seems to have a protective role in mental health, as it reduces anxiety and depression (22) and decreases the possibility of psychological distress. In the general population, some differences are noted: women usually report higher social support levels than men (23), with a greater socioeconomic situation that contributes to higher perceived social support (24). In the field of addiction, social support seems to be a protective factor, too. For alcohol-dependent people, social support perceived by friends and partners prevent risks against relapse, and for MacDonald (25), the higher the social support (i.e., number of individuals and quality of social support), the more abstinence is successful. A higher social support is predictive to an earlier onset of care, less relapse, and peers contribute to better emotion regulation.

Several studies looked at the link between social support and gambling. In a meta-analysis in adolescents and young adults' gamblers, social support appears to be a protective dimension of gambling addiction (26). Indeed, young problematic gamblers report having a lower social support (27, 28). Among adult gamblers, studies show a strong relationship between social support and problematic gambling (29). More precisely, problematic male gamblers tend to report less social support than occasional gamblers (30). With problem gamblers on treatment, social support is positively correlated with treatment success (21), gambling abstinence, and lower relapse rates (31). Social support can also be found among fellow players. Conversely, several studies indicate a lower prevalence among older people compared to younger (32). A study carried out among a population of older people living in a rural place shows that the more people gambled around tables, the more they reported having strong and quality social support (33). We emphasize here that social support is an individual variable in that it refers to an individual's perception of the quality and satisfaction with the social support received.

Eventually, since the 2000s, personality traits and pathological gambling have been extensively studied (34, 35). Pathological gamblers appear to have, on average, lower Consciousness and Agreeableness scores, and a higher Neuroticism score (34). In addition, other studies highlight a lower opening in non-pathological gamblers (36–38). Differences are noted between the type of game involved and personality traits. People who invest in card games, bingo, or dice games have higher levels of Extraversion and Agreeableness compared to other gamblers. People with lower agreeableness scores invest more in solitaire games such as slots or the lottery, which requires less social interaction (39). Furthermore, the ability to associate stimuli and form judgments about them depends in part on the participant's personal traits (40). The links between personality traits and the impact of advertising have been little investigated in the scientific literature. Nevertheless, insofar as certain personality traits are associated with a greater propensity to react negatively to stimuli and to feel these negative emotions (i.e., neuroticism), it is likely that this emotional feeling will influence the perception and memory encoding of stimuli. Similarly, because evaluative learning (i.e., the formation of judgments toward a neutral object) depends primarily on contingency awareness, i.e., the ability to detect the co-occurrence of stimuli and associate them in memory [see (41) for a review], neuroticism is expected to play a central role in the recognition and recall of advertisements. As a first step in this direction, we focused on the impact of the Five-Factor Model on advertising influence.

### Study Rationale

Overall, studies investigated the social support in a population of pathological gamblers with low perceived social support. Because emotion regulation and physiological stress is a modulator of executive functioning via its influence on vagal tone (42), stress, and emotion regulation can impact the memorization of advertising messages, and the perception of their content: individuals who are more vulnerable to stress are more likely to perceive messages including a relevant, gambling-related, stimulus, and show better memorization of these messages. Since social support improves emotion regulation, it can be assumed that better social support will lead to better emotion regulation and thus to reduced sensitivity to appetitive, advertising stimuli. A similar reasoning can be made about personality traits, which are involved in emotion regulation [see (43) for a review].

We focus mainly on young people and pathological gamblers who have started a therapeutic protocol. The present study is intended to capture social support among a variety of gamblers looking at problematic gamblers vs. none and looking at online vs. offline gamblers or both. Additionally, the multiplicity and diversity of protocols evaluating the impact of gambling campaigns complicates the understanding of this phenomenon. Through an original protocol using campaign slogans disseminated in 2018, the objective of this study is to understand the way in which gambling campaigns influence recall, incentive, and gambling behavior. Furthermore, we looked at personality traits across a diversity of sociodemographic and psychological variables that will increase knowledge in this domain. Hence, in this study, we first hypothesize that pathological gamblers perceived lower social support than moderate, or no risk gamblers, and online and mixed gamblers perceived lower social support than offline gamblers. We also expect that pathological gamblers show a higher score of Consciousness and Agreeableness and a lower score of Extraversion than no risk gamblers. Second, we expect a greater recall, incentive, and behavior intentions after watching or hearing an advertising campaign for (i) severe-risk gamblers vs. non-risk gamblers and (ii) for online and mixed gamblers vs. offline gamblers.

## Methods

### Participants

Participants were recruited through the social media site Facebook and online gambling forums (poker-academie.com, clubpoker.net, communaute-forum.pmu.fr). Participants were required to be 18 years of age or older, have gambled at least one time in the 12 last months, and lived in France during that period. Excluded from the study were people who did not speak French. One hundred fourteen adults were recruited. Five respondents were excluded because three did not gamble for the last 12 months and two did not live in France for the last 12 months. Analyzes were conducted on 109 (77 men, 32 women). Participants are 35.8 years old on average (SD = 11.9). All demographics are reported in Table 1. Participants completed the study online.

**Table 1:** Participants demographics.

	***N* (%)**
**Gender**	
Men	77 (70.6)
Women	32 (29.4)
**Level of education**	
None	1 (0.9)
Under high school diploma	10 (9.1)
high school diploma or similar	23 (21.1)
high school diploma more 2 or 3 years	32 (29.3)
high school diploma more 4 years	43 (39.4)
**Living space**	
Own housing	91 (83.5)
To friends or family	16 (14.7)
To institution	2 (1.8)
**City size**	
Very small city (<5,000 citizens)	29 (26.6)
Small city (between 5,000 and 20,000 citizens)	24 (22)
Medium city (between 20,000 and 50,000 citizens)	16 (14.7)
Big city (more than 50,000 citizens)	40 (36.7)
**Age**	Mean (standard deviation)
	35.8 (11.9)

### Procedure

Before accessing the questionnaires, participants were informed of the study objectives, the academic framework in which it is registered, the criteria for inclusion and exclusion, the anonymity of the information collected, and the possibility of stopping the filling at any time without any information being recorded. An email address has been created to answer participants' questions and disseminate results of the study. Once informed of the procedure, subjects agreed to participate in the study and began filling out the questionnaires. The study took around 15 min to complete. The data were collected between February and March 2019.

### Self-Reported Measures

#### Canadian Problem Gambling Index (α = 0.84)

We used the French version of the Canadian Problem Gambling Index (CPGI) to assess participants' level of gambling problems [nine items, (44)]. Participants answered on a four-point Likert scale being 0 (never) to 3 (almost always). In this study, participants were categorized in three categories: “non-risk gambler,” “moderate-risk gambler,” and “severe-risk gambler” (i.e., pathological gamblers).

#### Big Five Inventory—French Version

The Big Five Inventory—French Version (BFI-FR) scale contains 45 items that allow the five dimensions of personality to be assessed. To answer these questions, a five-point Likert scale is proposed ranging from 1 (strongly disapproves) to 5 (strongly approves)^1^.

#### Social Support Questionnaire

The short version of the Social Support Questionnaire 6 (SSQ6) scale was used. The validated French version (46) aims at evaluating the resources of one's support network and its perceived adequacy. Participants indicated (i) the initials of the resource people (nine people maximum), then (ii) the quality of the relations with these people on a Likert scale going from 1 to 6 (very dissatisfied to very satisfied). We computed two scores: social network availability (i.e., the number of people that the individual questioned identifies, from 0 to 54) and an adequacy score (i.e., sum of the adequacy scores obtained, from 0 to 36). Both dimensions had excellent psychometric qualities (α_Availability_ = 0.90, α_Availability_ = 0.93).

#### Impact of Gambling Advertisement

An *ad hoc* questionnaire has been created to assess the impact of gambling advertisements. We selected nine slogans of three different game operators disseminated online and in public spaces in 2018 in France. Two false slogans had been included into the list. Each one of these slogans were presented to participants to evaluate their recall with two items: 0, “I don't remember,” and 1, “I remember.” The sum of these scores provides an average recognition index ranging from 0 to 11. When participants recalled seeing an advertisement, they were (i) asked to recall the name of the game operator that disseminated the slogan (correct answer = 1, wrong answer = 0). They were then asked (ii) whether they wanted to play after watching or listening (incentive score, binary, 0 or 1). We computed a binary incentive score and behavior score (each coded 0 and 1).

#### Sociodemographic and Gamble Practices

Participants indicated their gender, age, employment situation, highest level of education, place of residence, size of city of residence, and country of residence. An additional question was added to assess the age of gambling onset.

### Analytic Strategy

Analyses were conducted using RStudio and JASP. Analyses have been conducted as follows. Following recent recommendations by **(author?)** (47), we conducted analyses following a Bayesian approach in addition to the classical frequentist approach. Bayesian analyses allow testing for the likelihood of either the alternative or the null hypothesis, hence distinguishing data showing no clear evidence whatsoever from data supporting the null hypothesis (48, 49). The Bayes factor (BF) compares the probability of the data under one model to that under another and provides evidence in favor of either the null hypothesis (BF_01_) or the alternative hypothesis [BF_10_; (50, 51)]. Inclusion BFs for the moderating effect of the number of persons available for social support and satisfaction regarding social support scores are reported across matched models. The Inclusion BF reflects the evidence for all models with a particular term, compared to all models without this particular term. For these analyses, Cauchy's prior was first set to 0.35, which means that 50% of the values from the prior distribution are comprised between *r* = 0.35 and −0.35. All analyses were conducted on JASP 0.14 (JASP Team, 2017).

We first conducted a multinomial regression model with the categories of gambler as the outcome and social support scores (availability and adequacy), personality scores, gender, age, diploma, type of housing, size of the city, and whether they started to play as a minor as predictors (model 1, see Table 2 for all estimates). We then conducted a set of one multiple linear regression and two multiple ordinal regression model with categories of gambler as predictors recognition scores (model2a), incentive scores (model2b), and behavior scores (model2c), and categories of gambler, type of gambling (offline vs. online and mixed gamblers), social support scores (availability and adequacy), personality scores, gender, age, diploma, type of housing, size of the city, and whether they started to gamble as a minor as predictors (see Table 3 for all estimates). We report results from analyses conducted with the classical, frequentist approach, and BFs and Inclusion BF.

**Table 2:** Multinomial logistic regression.

**Model fit measures**						
				**Overall model test**		
**Model**	**Deviance**	**AIC**	***R***^2^ **McF**	***χ*** ^2^	***df***	***p***
1	157.28	221.28	0.21198	42.307	30	0.067

Table 3Multiple regression with categories of gamblers as predictors recognition socials, intention and behavior.

**Table:** 

**Model 2a. ANOVA omnibus tests**					
	***SS***	***df***	***F***	***p***	***η** ^2^_***p***_*
Model	58.2232	17	1.59611	0.083	0.242
Age	11.2199	1	4.20057	0.043	0.074
Categories of gamblers	1.4490	1	0.54247	0.463	0.018
Mode of gambling	2.3944	1	0.89642	0.346	0.017
Gender	0.0157	1	0.00589	0.939	0.006
Diploma	8.1696	1	3.05856	0.084	0.017
House	0.1294	1	0.04845	0.826	0.005
Work	1.9558	1	0.73224	0.395	0.008
kind_residence	1.2153	1	0.45497	0.502	0.005
size_city	3.3642	1	1.25949	0.265	0.014
gamble_less18y	9.2900	1	3.47804	0.066	0.059
Availability	11.7944	1	4.41564	0.039	0.046
Satisfaction	0.7913	1	0.29627	0.588	0.006
Openness	3.4801	1	1.30291	0.257	0.016
Consciousness	0.0879	1	0.03291	0.856	0.000
Extraversion	0.7189	1	0.26913	0.605	0.003
Agreeability	2.1469	1	0.80378	0.372	0.010
Neuroticism	3.94e−4	1	1.47e−4	0.990	0.000
Residuals	227.0389	85			
Total	285.2621	102			
**Model 2b. Binomial logistic regression**					
**Model Fit Measures**					
**Model**	**Deviance**	**AIC**	***R***^2^ **McF**		
1	54.522	94.522	0.36232		
**Predictor**	**Estimate**	***SE***	***Z***	***p***	
Intercept	−2.7041984	5.615389	−0.481569	0.630	
Age	−0.0719102	0.053545	−1.342978	0.179	
Categories of gamblers					
Non-risk–moderate risk	0.6711522	0.931857	0.720231	0.471	
Non-risk–severe risk	2.6489519	1.316277	2.012458	0.044	
Mode of gambling					
Outline–online	−1.0152507	1.224167	−0.829340	0.407	
Outline and online–outline	0.2604371	0.977093	0.266543	0.790	
Gender	−0.5510260	1.185495	−0.464807	0.642	
Diploma	0.0475954	0.344133	0.138305	0.890	
House	0.2793072	0.877094	0.318446	0.750	
Work	0.1249741	0.252195	0.495545	0.620	
kind_residence	−0.6298236	1.510053	−0.417087	0.677	
size_city	−0.0269201	0.366966	−0.073359	0.942	
gamble_less18y	−0.8894833	0.897306	−0.991282	0.322	
Availability	0.0137726	0.049983	0.275544	0.783	
Satisfaction	0.0618115	0.076301	0.810100	0.418	
Openness	−0.0065253	0.081765	−0.079806	0.936	
Consciousness	0.1114438	0.086190	1.292998	0.196	
Extraversion	−0.0154560	0.071490	−0.216197	0.829	
Agreeability	−0.0846025	0.076920	−1.099869	0.271	
Neuroticism	0.1162461	0.072626	1.600616	0.109	
**Model 2c. Classical regression/ANOVA**					
**Model results**					
**Loglikelihood ratio tests**

**Table:** 

**Model Fit Measures**						
			**Overall Model Test**			
**Model**	**Deviance**	**AIC**	${\text{R}}_{McF}^{2}$	**X** ^**2**^	**df**	**p**
1	78.9	119	0.243	25.3	19	0.150

**Table:** 

**Model Coefficients - cptt_score_bin**									
	**95% Confidence Interval**						**95% Confidence Interval**		
**Predictor**	**Estimate**	**Lower**	**Upper**	**SE**	**Z**	**p**	**Odds ratio**	**Lower**	**Upper**
Intercept	−2.89749	−11.15186	5.3569	4.2115	−0.6880	0.491	0.0552	1.43e-5	212.060
Online Gambling	0.38849	−0.49538	1.2724	0.4510	0.8615	0.389	1.4748	0.6093	3.569
Gender	0.22221	−1.42614	1.8706	0.8410	0.2642	0.792	1.2488	0.2402	6.492
Diploma	−0.02347	−0.51666	0.4697	0.2516	−0.0933	0.926	0.9768	0.5965	1.600
Housing	0.60371	−0.72969	1.9371	0.6803	0.8874	0.375	1.8289	0.4821	6.939
Work	−0.10551	−0.50083	0.2898	0.2017	−0.5231	0.601	0.8999	0.6060	1.336
Residence	−0.18582	−1.80701	1.4354	0.8272	−0.2246	0.822	0.8304	0.1641	4.201
City Size	0.25600	−0.31775	0.8297	0.2927	0.8745	0.382	1.2918	0.7278	2.293
Gambling as Minor	−1.56276	−3.02669	−0.0988	0.7469	−2.0923	0.036	0.2096	0.0485	0.906
Social Support Availability	0.06964	0.00245	0.1368	0.0343	2.0313	0.042	1.0721	1.0024	1.147
Social Support Satisfaction	−0.04720	−0.13636	0.0419	0.0455	−1.0378	0.299	0.9539	0.8725	1.043
BFI-Openness	0.00314	−0.10635	0.1126	0.0559	0.0562	0.955	1.0031	0.8991	1.119
BFI-Conscientiousness	0.07675	−0.04518	0.1987	0.0622	1.2337	0.217	1.0798	0.9558	1.220
BFI-Extraversion	0.05520	−0.05080	0.1612	0.0541	1.0207	0.307	1.0568	0.9505	1.175
BFI-Agreableness	−0.04567	−0.17632	0.0850	0.0667	−0.6852	0.493	0.9554	0.8383	1.089
BFI-Neuroticism	−0.08950	−0.19295	0.0139	0.0528	−1.6957	0.090	0.9144	0.8245	1.014
Typology of gamblers:									
Moderate Risk Gamblers-No-Risk Gamblers	1.02460	−0.56869	2.6179	0.8129	1.2604	0.208	2.7860	0.5663	13.707
Severe Risk Gamblers-No-Risk Gamblers	2.40168	0.29316	4.5102	1.0758	2.2325	0.026	11.0418	1.3407	90.941
Age	0.01208	−0.05463	0.0788	0.0340	0.3550	0.723	1.0122	0.9468	1.082
rgp typ jeu	0.81953	−0.87463	2.5137	0.8644	0.9481	0.343	2.2694	0.4170	12.350

*Estimates represent the log odds of “cptt_score_bin = 1” vs. “cptt_score_bin = 0.”*

## Results

### Effect of Social Support and Personality on Categories of Gamblers

**Figure 1:**
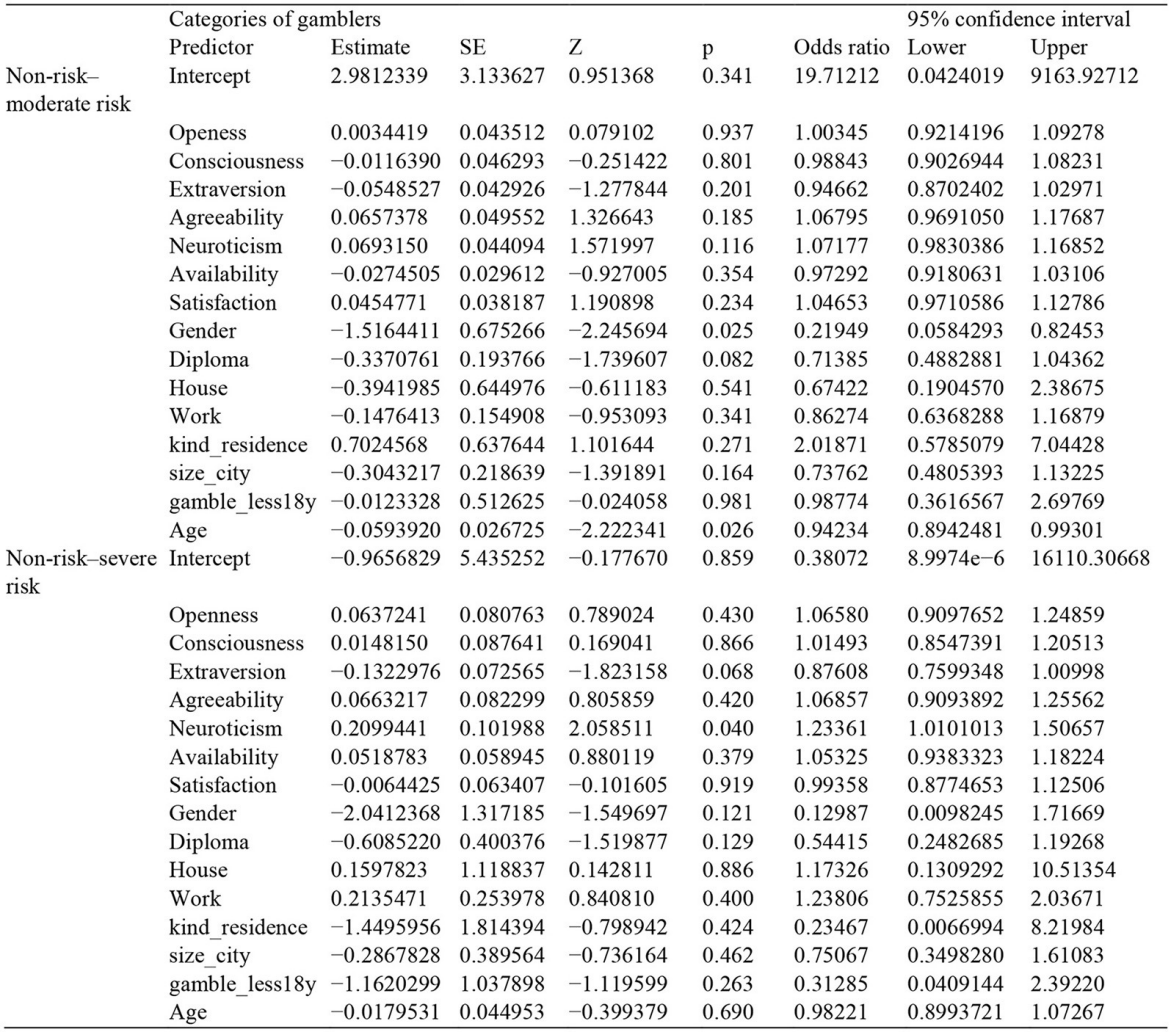
Differences between categories of gamblers in terms of demographics and psychologic questions.

Model 1 was overall marginally significant, $\chi_{\left(30\right)}^{2}$ = 42.3, *p* = 0.06, Akaike information criterion (AIC) = 221, *R*^2^ McF = 0.212. Model 1 revealed a main effect of neuroticism on categories of gamblers, $\chi_{\left(2\right)}^{2}$ = 7.02, *p* = 0.03. We did not find a significant difference between “non-risk” and “moderate-risk” gamblers, odds ratio (OR) = 1.07, standard error (SE) = 0.044, *p* = 0.11. However, we found a significant difference for “non-risk” and “severe-risk” gamblers, such that gaining one point on the neuroticism scale leads to a 23% increase in being in the “severe-risk” category, OR = 1.23, SE = 0.01, *p* = 0.04 (see Figure 1). We found a main effect of gender, $\chi_{\left(2\right)}^{2}$ = 6.80, *p* = 0.033. We found a significant difference in gender between “non-risk” and “moderate-risk” gamblers, such that being a man leads to a 79% increase of being in the “moderate-risk” category OR = 0.21, SE = 0.67, *p* = 0.025. No other effect was significant (*p* < 0.097).

Bayesian analyses showed that the model including age, neuroticism, gender, and diploma yielded the strongest evidence for the alternative hypothesis compared to all other models, BF_10_ = 72.71. Inclusion BF showed small evidence for the alternative hypothesis for neuroticism, BF_Inclusion_ = 2.92, gender, BF_Inclusion_ = 3.08 and diploma, and BF_Inclusion_ = 2.31.

### Effect of Categories of Gamblers on Advertisement Recognition (Model2a)

Model2a was overall marginally significant, *F*_(17, 85)_ = 1.60, *p* = 0.083, η^2^_*p*_ = 0.23. The analysis revealed a significant main effect of social support adequacy, *b* = −0.05, 95% CI [−0.10, 0.001], *t*_(85)_ = −1.94, *p* = 0.039, η^2^_*p*_ = 0.064 so that lower adequacy predicted higher recognition. We also found a significant main effect of age, *b* = −0.03, 95% CI [−0.06, −0.001], *t*_(85)_ = −2.11, *p* = 0.043, η^2^_*p*_ = 0.075, such that younger participants had higher recognition scores. Eventually, we found a marginally significant main effect of onset of gambling, *b* = −0.66, 95% CI [−1.39, −0.06], *t*_(85)_ = −1.82, *p* = 0.06, η^2^_*p*_ = 0.039, such that the earlier the onset of gambling, the higher the recognition scores. We did not find any other effect (*p*s <0.07).

Bayesian analyses showed that the model including age, social support adequacy, and onset of gambling yielded the strongest evidence for the alternative hypothesis compared to all other models, BF_10_ = 92.71. Inclusion BF showed substantial evidence for the alternative hypothesis for age, BF_Inclusion_ = 5.19; onset of gambling, BF_Inclusion_ = 5.11; and anecdotal evidence for diploma, BF_Inclusion_ = 2.

### Effect of Categories of Gamblers on Perceived Incentive to Play (Model2b)

Model2b was overall significant, $\chi_{\left(19\right)}^{2}$ = 31, *p* = 0.04, AIC = 94.5, *R*^2^ McF = 0.362. Model2b revealed a marginally significant main effect of the category of gambler, $\chi_{\left(2\right)}^{2}$ = 4.65, *p* = 0.09. We did not find a significant difference between “non-risk” and “moderate-risk” gamblers, OR = 1.95, SE = 0.93, *p* = 0.47 on perceived incentive. However, we found a significant difference for “non-risk” and “severe-risk” gamblers, such that being in the “severe-risk” category leads to a 1,400% increase in feeling incented to gamble, OR = 14.13, SE = 1.31, *p* = 0.044. No other effect was significant (*p* < 0.097).

Bayesian analyses showed that the model including only the category of gamblers factor yielded the strongest evidence for the alternative hypothesis compared to all other models, BF_10_ = 111.41. Inclusion BF showed strong evidence for the alternative hypothesis for category of gamblers, BF_Inclusion_ = 17.23.

### Effect of Categories of Gamblers on Intention to Play (Model2c)

**Figure 2:**
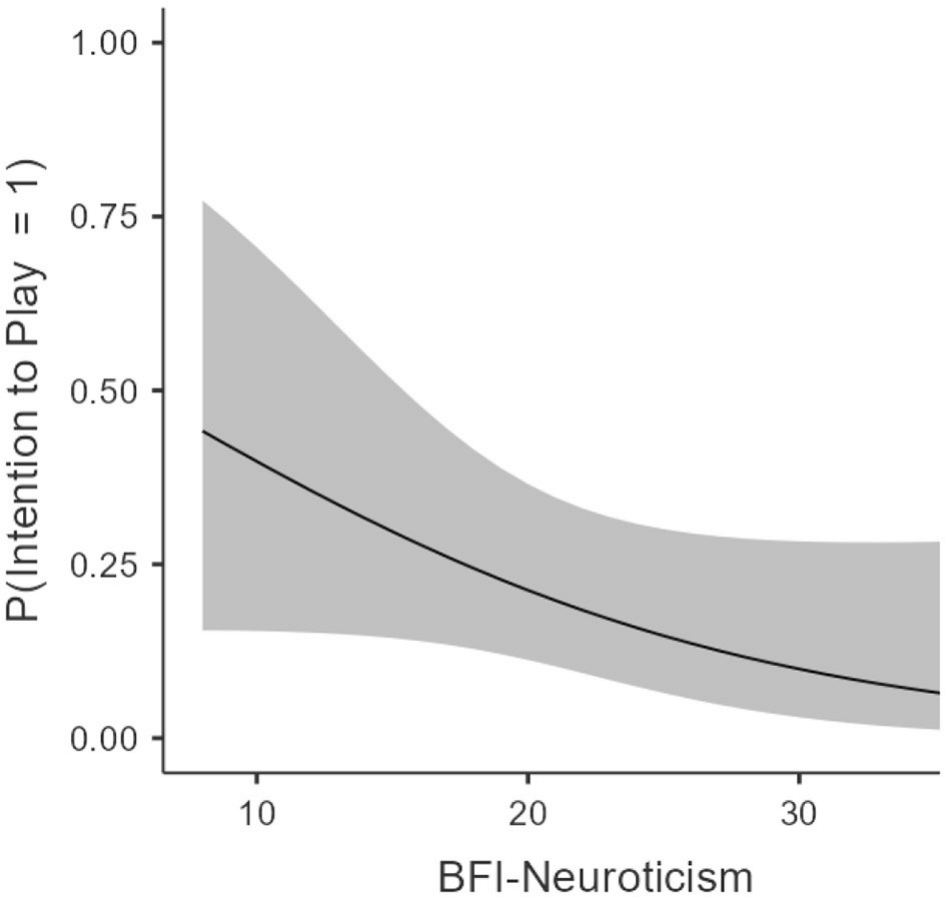
Estimated Marginal Means between neuroticism and intention to play.

Model2c was overall not significant, $\chi_{\left(19\right)}^{2}$ = 25.3, AIC = 119, *R*^2^ McF = 0.243. The analysis revealed a significant main effect of social support availability, $\chi_{\left(1\right)}^{2}$ = 4.37, OR = 1.07, SE = 0.03, *p* = 0.042 such that higher availability led to lower intention to play. We also found a significant main effect of the onset of gambling, such that a decrease of 1 year in the onset lead to an 80% increase in probability of reporting an intention to play after seeing an advertisement, OR = 0.21, SE = 0.74, *p* = 0.036. We also found a marginally significant effect of categories of gambler, $\chi_{\left(1\right)}^{2}$ = 5.27. We did not find any significant difference between “non-risk” and “moderate-risk” gamblers, OR = 2.78, SE = 0.81, *p* = 0.20, but found a significant difference between “non-risk” and “severe-risk” gamblers, OR = 11.04, SE = 1.07, *p* = 0.026, such that being in the “severe-risk” group led to a 1,100% increase in probability of reporting having the intention to play. Eventually, we found a marginally significant effect of neuroticism, $\chi_{\left(1\right)}^{2}$ = 3.06, OR = 0.91, SE = 0.05, *p* = 0.09, such that, surprisingly, a decrease of one point in neuroticism lead to a 10% higher probability of having the intention to play (see Figure 2).

Bayesian analyses showed little convincing evidence for any model. The model including onset of gambling, conscientiousness, social support availability factor, and category of gamblers yielded only moderate evidence for the alternative hypothesis compared to all other models, BF_10_ = 9.87. Inclusion BF showed acceptable evidence for the alternative hypothesis for the category of gamblers, BF_Inclusion_ = 5.61, and anecdotal evidence for onset of gambling, BF_Inclusion_ = 2.8.

## Discussion

This study investigated and compared how variables related to self-regulation, such as social support, dimensions of personality predicted perception, and memorization of advertising campaigns, and were associated with problem gambling among severe-, moderate-, and non-risk gamblers and associated with online gambling (i.e., sport and poker). The present protocol is based on the recall of different slogans diffused by French Gambling operators for the last 12 months before the study, the perceived encouragement to gamble, and the behavior. Overall, although some of our results are only marginally significant, there seems to be an effect of the variables associated with self-regulation (i.e., neuroticism and social support) on ad recognition, perceived incentive to play, and intention to play.

### Does Gambling Severity Change Advertising Influence?

Notably, when the onset of gambling is before 18 years old, participants had more chances to recall more gambling advertisements when they were adults. Although the onset of gambling was not the primary hypothesis, this variable appears significant in our campaigns recall model. Several studies with adolescents and adults show a correlation between when the onset of gambling and problematic gambling (52, 53).

Overall, severe-risk (i.e., pathological) gamblers seem to be more prone to gamble after watching or hearing a campaign than the others. These results are both concordant and discordant with the literature. Regarding recall, some studies do not find effect among gamblers (10, 54), whereas others do (12). Concerning intentions and behavior, our results are similar to the literature. Different ads impact the intention of gambling and the behavior particularly for problematic gamblers (12, 55). Our results indicate the lack of relationships between the recall of gambling advertising and online gambling, whereas some research indicates that the exposure to campaigns is more important for online gamblers (55). All these elements indicate the absence of longitudinal and experimental studies and valid tools. Interestingly, there was no strong link between social support perceived and typology of gamblers.

### Is Advertising Influence Different Depending on Personality Traits?

We also found that among personality traits, neuroticism appeared to have the most robust impact on the perception and recall of advertisements, and the propensity to treat them as “appetitive” stimuli. This is not surprising insofar as neuroticism is associated not only with more frequent experience of negative emotions but also with a weaker ability to regulate these emotions. Although negative emotion regulation and neuroticism are distinct constructs, they nevertheless overlap to some extent, with neuroticism being associated with extraversion, in contrast to emotion regulation (56). A surprising finding is that neuroticism appears to be negatively associated with play, implying that the play stage is likely associated with positive emotions—and reinforcement. About personality traits, our results are not completely in line with the literature, as severe-risk gamblers presented higher levels of Neuroticism compared with “recreational” gamblers but did not present low scores of Conscientiousness and Agreeableness. We suggest that future studies should explore if subgroups of gamblers (e.g., online vs. offline, gamblers with morbidity vs. not) change regarding personality traits.

### Does Social Support Hinders Advertising Influence?

Moreover, social support is significantly lower among online gamblers compared to offline gamblers, but no association was found between social support and type of gamblers. This finding is contrary to expectations but similar with few studies. A systematic review on psychosocial risks for gambling and problem gambling in Nordic countries, Nordmyr and Forsman (57) indicate that if social support could be a protective factor of problematic gambling but not in all studies but in two studies, social support is not associated with problematic gambling to young people (12, 58)].

In addition, social support is closely related not only to social network but also to loneliness (24). Family and peers may be protective factors of pathological gambling; more studies should assess what kind of support gamblers and particularly online gamblers defined as supporting past the adequacy of social support. Surprisingly, few studies focus on the effect of isolation on social and addictive behaviors, and consequently on gambling, even though it is a central variable in the study of social behavior in animal models, as the ability to voluntarily isolate oneself may allow for better management of daily stress (59). Future studies should address this issue in more detail.

## Limitations

However, our conclusions are somewhat hindered by our relatively small sample size, which may explain some of our marginally significant results. Specifically, with social scale support, several participants canceled their answers due to the length of the questionnaire. Moreover, the number of responses tended to decrease between the first one answer and the last one.

## Conclusion

This study is a unique contribution for several reasons. First, we used original memorization measures involving long-term memory rather than immediate recall. Second, we identified novel factors related to self-regulation that may be crucial in understanding how gamblers interact with their social environment and regulate their gambling behaviors. These first results pave the way for potential therapeutic management processes, particularly in the context of systemic therapies that take charge of the individual through his interactions with his social environment. Encouraging gamblers, initially, to shift their practices toward games where social interactions exist could allow low-addicted gamblers to avoid seeing their situation worsen. Gamblers in a more serious situation may also benefit from this type of approach. A second step would be to offer help and better social support to severely affected gamblers. This could be done, for example, by offering help—professionally, or via their social network—automatically triggered via smartphone when the gambler is exposed to or near stimuli that can trigger gambling behavior. Focusing on the social—and societal—aspect of advertising could help mitigate these effects. Eventually, on the other hand, the lack of a standardized protocol multiplies the development of new, non-validated methods.

## Data Availability Statement

The raw data supporting the conclusions of this article will be made available by the authors, without undue reservation.

## Ethics Statement

Ethical review and approval was not required for the study on human participants in accordance with the local legislation and institutional requirements. The patients/participants provided their written informed consent to participate in this study.

## Author Contributions

All authors have read and approved the manuscript for submission to Frontiers in Psychiatry: Addictive Disorders: have made a substantial contribution to the conception, design, gathering, analysis and/or interpretation of data and a contribution to the writing and intellectual content of the article: and acknowledge that they have exercised due care in ensuring the integrity of the work.

## Conflict of Interest

Laboratory of LR received the support of La Française des Jeux, through a Scientific Interest Group (GIS) Jeu et Société (Play and Society) with four French universities Université Paris Descartes, Université de la Sorbonne, Université Paris 13, and Université Paris Ouest Nanterre La Défense. The remaining authors declare that the research was conducted in the absence of any commercial or financial relationships that could be construed as a potential conflict of interest.

## Publisher's Note

All claims expressed in this article are solely those of the authors and do not necessarily represent those of their affiliated organizations, or those of the publisher, the editors and the reviewers. Any product that may be evaluated in this article, or claim that may be made by its manufacturer, is not guaranteed or endorsed by the publisher.

## Footnotes

^1^A score is calculated for each dimension by averaging the items. This scale has good internal validity (α = Openness = 0.72, Conscientiousness = 0.79, Extraversion = 0.81, Agreeableness = 0.66, Neuroticism = 0.83), a consistent factor structure and a good distribution of items consistent with the initial American version (45).
